# Acute Epigallocatechin 3 Gallate (EGCG) Supplementation Delays Gastric Emptying in Healthy Women: A Randomized, Double-Blind, Placebo-Controlled Crossover Study

**DOI:** 10.3390/nu10081122

**Published:** 2018-08-20

**Authors:** Renata C. Fernandes, Vanessa A. Araújo, Bruna M. Giglio, Ana Clara B. Marini, João F. Mota, Kim-Ir-Sen S. Teixeira, Paula A. Monteiro, Fabio S. Lira, Gustavo D. Pimentel

**Affiliations:** 1 Clinical and Sports Nutrition Research Laboratory (Labince), Faculty of Nutrition, Federal University of Goiás, Goiânia, Goiás 74605-080, Brazil; renata_cfernandes@hotmail.com (R.C.F.); vanenut95@gmail.com (V.A.A.); brunamgiglio@gmail.com (B.M.G.); ac.marini22@gmail.com (A.C.B.M.); jfemota@gmail.com (J.F.M.); 2 Department of Radiology and Diagnostic by Imaging, Medicine University, Federal University of Goiás, Goiânia, Goiás 74605-050, Brazil; kimirsen@terra.com.br; 3 Exercise and Immunometabolism Research Group, Department of Physical Education, Paulista State University, Presidente Prudente, São Paulo 19060-900, Brazil; paulinha__1003@hotmail.com (P.A.M.); fabioslira@gmail.com (F.S.L.)

**Keywords:** *Camellia sinensis*, EGCG, catechin, gastric emptying, satiety response, satiation

## Abstract

**Background**: Epigallocatechin 3 Gallate (EGCG) appears to act in appetite control through hormonal modulation. However, there is a lack of elucidation of EGCG’s action mechanisms, especially in humans. The aim of this study was to evaluate the effects of acute EGCG supplementation on gastric emptying and its relation to blood hormones, glucose and appetite perceptions in healthy women. **Methods**: 22 healthy adult women were included in a randomized, double-blind, placebo-controlled crossover study. On two separate occasions, 1 week apart from each other, we offered 800 mg of corn starch (placebo) or 752 mg of EGCG. Appetite was assessed through gastric emptying; perceptions of hunger, desire to eat and satiation; and plasma insulin, adiponectin, leptin and glucose concentrations. The evaluations were carried out in fasting, 30, 90 and 150 min after supplementation. **Results**: EGCG supplementation induced higher relative gastric volume at 30 and 90 min. Satiation at 90 min was higher in the EGCG group. Adiponectin concentrations at 150 min were higher with EGCG, but no difference was found for glucose, insulin and leptin concentrations. **Conclusions**: Acute EGCG supplementation is able to delay gastric emptying in healthy women to a small, but statistically significant extent. This study was registered at the Brazilian Registry of Clinical Trials (ReBEC) as RBR-9svwrv.

## 1. Introduction

Overweight treatment has gained prominence in recent decades, especially in relation to food intake control alternatives. Studies with the use of bioactive compounds, including catechins present in green tea (*Camellia sinensis*), appear in the literature as control strategies for body weight [1,2,3,4]. The mechanism by which green tea works to reduce body weight has not been fully elucidated. Studies have suggested that the anti-obesity effects are due to Epigallocatechin 3 Gallate (EGCG), the main flavonoid present in green tea [5,6,7]. In addition, research has hypothesized that EGCG acts in appetite control through hormonal modulation [5,6].

Kao, Hiipakka and Liao [5] found that intraperitoneal EGCG injection in rodents (>98% pure) for 7 days decreased food intake and body weight, plasma leptin, insulin, triacylglycerol, total cholesterol and glucose concentrations. Moreover, the effects were similar in leptin receptor gene-deficient obese mice, demonstrating that the beneficial effects of EGCG on appetite occur regardless of changes in this receptor. It was recently demonstrated in vitro (Caco-2 cells) and ex vivo (intestinal cells in rats) that the treatment with EGCG was able to induce the secretion of anorectic hormones, such as peptide YY, cholecystokinin and glucagon-like peptide (GLP-1) [6].

Assessment of potential physiological and hormonal mechanisms involved in acute EGCG supplementation is essential in the search for nutritional strategies that may reduce appetite and food intake in the short term, controlling the body weight. However, studies with isolated EGCG, especially in humans, are scarce, and the mechanisms of action regarding control of appetite have not been fully expounded.

The aim of this study was to evaluate the effects of acute EGCG supplementation on gastric emptying and its relation to blood hormones, glucose and appetite perceptions in healthy women. Considering that gastric emptying was defined as the primary end-point, we hypothesized that acute EGCG supplementation could modulate appetite by slowing down gastric emptying and increasing secretion of anorectic hormones in healthy women.

## 2. Materials and Methods

### 2.1. Design and Ethical Aspects

The study design was a randomized, double-blind, placebo-controlled, crossover clinical trial. The study was approved by the Research Ethics Committee of the Federal University of Goiás, protocol number 1.985.617 and registered at the Brazilian Registry of Clinical Trials (ReBEC) as RBR-9svwrv. All participants received information about the research and signed the informed consent form before their inclusion in the study. All procedures adopted are in accordance with the Declaration of Helsinki 1975, as revised in 1983.

### 2.2. Participants

Twenty-two healthy young adult women of Latin American and European descent participated in this study. Only healthy individuals were chosen, in order to obtain an initial knowledge of the possible mechanistic actions of EGCG on appetite. All volunteers had a stable weight in the last 12 months (weight gain or loss less than 5%). Excluded from the study were women of Asian descent with a body mass index (BMI) outside the normal range according to WHO classification [8] (<18.5 kg/m^2^ or >24.9 kg/m^2^); women with autoimmune diseases or taking immunosuppressive drugs; women with clinical diagnosis of diabetes, thyroid dysfunction, chronic kidney disease or liver disease; women who had undergone bariatric surgery; women who were chronic alcoholics or smokers; women who had taken hormonal medications (e.g., contraceptives) or appetite/body weight-management drugs (e.g., appetite suppressants) in the last 12 months; women who took proton pump inhibitor medications; and women who had participated in any food restriction program or had been using nutritional supplements during the past 12 months or who were lactose or fructose intolerant.

The number of subjects required for this study was based on a power calculation (80% power at 5% significance) using data from a previous study [9], in which the time to empty the stomach to 50% of initial volume was considered as the end-point primary, calculated and assuming a difference of 20.5 min, resulting in 16 participants. Thus, the sample size studied (22 participants) had sufficient power to detect statistically significant differences.

### 2.3. Protocol

The women were submitted to two tests with a 1-week interval (washout) [10]. In the EGCG group, the participants received two opaque capsules containing 94% EGCG (Teavigo^®^, Taiyo International, Minneapolis, MN, USA), weighing 376 mg per capsule, totaling 752 mg of EGCG. In the placebo group (control), women received two opaque capsules containing 400 mg of corn starch each, totaling 800 mg, with color and size similar to EGCG supplementation. The capsule packs were blinded by a person not involved in the research. Random assignment was performed by the simple blind draw of a person not involved in the data collection. Eleven volunteers were randomized to initiate the intervention with placebo and twelve with EGCG. One volunteer was excluded from the study after receiving EGCG. One week after the first test, the treatments were reversed.

**Figure 1:**
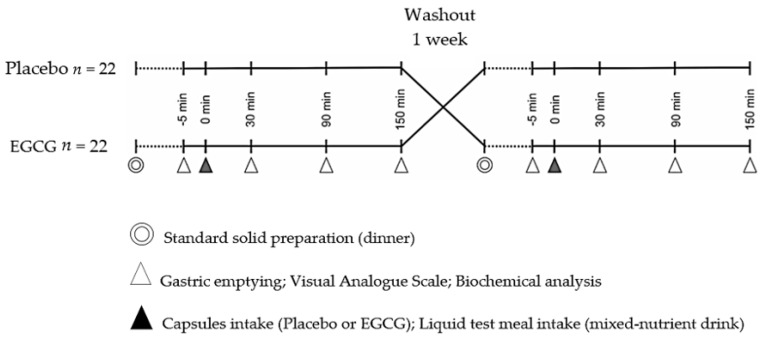
Study design.

Perceptions of hunger, desire to eat and satiation were rated by the Visual Analogue Scale (VAS). Plasma glucose and appetite regulating hormones (insulin, adiponectin and leptin) were evaluated by venous blood samples. Gastric volume was assessed by ultrasonography. All measurements were taken 5 min before supplementation and after that at 30, 90 and 150 min intervals leading up to post supplementation. A liquid test meal was given at the same time as the capsules (time 0 min) and consumed within 2 min, as shown in Figure 1.

Participants were instructed to ingest the EGCG or placebo capsules with the aid of the liquid test meal (295 mL of mixed-nutrient drink) within 2 min. The time of the mixed-nutrient drink intake was standardized to avoid discrepancies that could alter the values of gastric retention [9,11]. The macronutrient content of the test meal (Supplementary Table S1) was based on the mean nutrient intake at breakfast for all the participants.

The participants were instructed not to drink alcohol or beverages containing caffeine (coffee, cola and mate, black or green tea) for 72 h prior to the experiment and, on the previous day, not to perform physical activity and maintain the usual diet. Data collection was performed when the women were not in their menstrual period, and we chose to maintain the same weekday in the two collections in order to minimize the alteration in the previous day’s food intake.

The last meal prior to data collection (dinner) was standardized and delivered by researchers to avoid alteration in the production of appetite-related hormones on the day of the experiment. The volunteers were instructed regarding the time of ingestion to maintain 12 h of subsequent fasting. The nutritional composition of the standard solid preparation (dinner) was obtained by analyzing the mean usual intake reported by the participants at dinner and is presented in Supplementary Table S2.

### 2.4. Measures

#### 2.4.1. Body Composition and Habitual Food Intake Evaluation

Measurements of body weight, height and waist and hip circumferences were performed. We also performed a measurement of the sagittal abdominal diameter to characterize the central adiposity of the volunteers. The percentage of body fat was assessed using the Dual-energy X-ray absorptiometry (DXA) technique (Lunar DPX NT—DXA for Bone Health—GE Healthcare^®^, Australia, New Zealand).

The habitual food intake of the volunteers was evaluated by regular food recall collected by trained researchers. The calculation of total calories, macronutrients and total fibers was performed by Dietpro^®^ software (version 5.8, Minas Gerais, Viçosa, Brazil).

#### 2.4.2. Gastric Emptying Evaluation

Gastric emptying was assessed by ultrasonography, which is a safe, non-invasive and easily reproducible method. The technique allows accurate measurement of the retention rate and gastric contents elimination from the definition of each individual’s stomach volume [11]. A Logiq P6^®^ (GE^®^, Duluth, USA) device and a transducer with a convex probe with a frequency of 5 MHz were used. The procedure was previously standardized and performed by a radiology specialist.

The participants were directed to individual air-conditioned booths and evaluated in the supine position at four times: fasted (- 5 min) and after 30 min, 90 min and 150 min of EGCG or placebo capsules intake with the standardized liquid diet (mixed-nutrient drink), as presented in Figure 1. The participants remained seated, resting, between these time intervals.

The meal had to be liquid in consistency to facilitate the visualization of gastric contents by the ultrasound equipment. It was necessary to administer an anti-gases drug (Simethicone 40 mg) with 100 mL of water both 8 h and 1 h before the initial procedure to reduce gas interference in the stomach cavity and to reduce errors due to reading difficulties. This is a suitable procedure for measuring gastric emptying by ultrasonography, according to the technique described previously [11].

To evaluate the retained gastric volume, the probe was positioned in the epigastric region, in a longitudinal orientation, using the sagittal plane through the aorta and superior mesenteric vein [12]. The calculation was performed by the area of the ellipse formula given by π × A × B/4, where A is the longitudinal diameter; B is the anteroposterior diameter and π = 0.52 [11].

#### 2.4.3. Subjective Assessment of Appetite

Perceptions of hunger, desire to eat and satiation were performed by VAS, which provides an accurate way to predict appetite in young adults and has good reproducibility [13,14].

Three questions were asked: “Do you feel hungry right now?”, “Do you feel like eating right now?”, and “Does your stomach feel full right now?” The volunteers were instructed to make a point at the point where their hunger, desire to eat and fullness (full-stomach satiation) best represented their sensation at the time.

#### 2.4.4. Biochemical Analysis

Venous blood samples were collected into heparinized vacuum tubes at each time intervals. The blood was immediately centrifuged at 4000 rpm for 10 min at 10 °C in a refrigerated centrifuge (Hitachi Koki^®^, Tokyo, Japan) to separate plasma. Aliquots of plasma were immediately stored in a −80 °C freezer until analysis.

The blood glucose and insulin concentrations were determined by enzymatic calorimetric and immunoturbidimetry methods, respectively (ArchitectPlus^®^, Naperville, Illinois, USA). Adiponectin and leptin concentrations were assessed by the sensitive enzyme-linked immunosorbent assay (ELISA) kit (DuoSet, R&D systems^®^, Minneapolis, Minnesota, USA) according to the protocol described by the manufacturer.

### 2.5. Data and Statistical Analyses

The data was twice entered into Excel^®^ (Microsoft, Redmond, USA) spreadsheets. Data distribution was evaluated by the Shapiro-Wilk test. All variables were expressed as mean and standard error of the mean (SEM).

The VAS data variation for hunger sensation, desire to eat and satiation; the percentage and absolute retention; the calorie retention; and biochemical tests results (glucose, insulin, leptin and adiponectin) were analyzed by two-way ANOVA, adjusted for individual variation (crossover study), following the mathematical model: Y*_ijk_* = μ + α*_i_* + β*_j_* + γ*_k(j)_* + ε*_ijk_*; where Y*_ijk_* is the observation value obtained on individual *i*, at time *j*, under treatment k; μ is a constant; α*_i_* is the effect of individual *i*; β*_j_* is the effect of the evaluation time j; γ*_k(j)_* is the effect associated with treatment k, at time *j*; ε*_ijk_* is the residual effect (error) associated with the Y*_ijk_* observation.

The analysis of the area under the curve (AUC) and the variable ‘empty of calories per minute’ were analyzed by two-way ANOVA, adjusted for the individual variation (crossover study), following the mathematical model: Y*_ij_* = μ + α*_i_* + γ*_j_* + ε*_ij_*; where Y*_ijk_* is the value of the observation obtained in individual *i*, under treatment j; μ is a constant; α*_i_* is the effect of individual *i*; γ*_j_* is the effect of the evaluation treatment *j*; ε*_ij_* is the residual effect (error) associated with individual *i*, in treatment *j*.

Relative gastric retention (%) was calculated as a percentage at each time point. It was considered as 100% of the sum of the initial gastric volume of each participant with the 295 mL of the liquid test meal (mixed-nutrient drink) offered at time 0 min. The relative volumes retained at subsequent times were calculated in proportion to the time 0 min value. The retention of calories in the stomach was calculated considering the supply of 279.44 kcal from the mixed-nutrient drink with the volume (in mL) of the gastric content at time 0 min. The calories retained at other times were calculated in proportion to the initial value. The calorie emptying rate per minute was calculated by subtracting the initial calorie values in the stomach at the time 0 min (279.44 kcal) from that retained at the end of the study, at time 150 min, and divided by 150.

The level of significance was 5% (*p* < 0.05). Cohen’s d effect size was used to compare the means of the EGCG and placebo groups. All analyses were performed in R^®^ software under the supervision of a professional biostatistician.

## 3. Results

**Figure 2:**
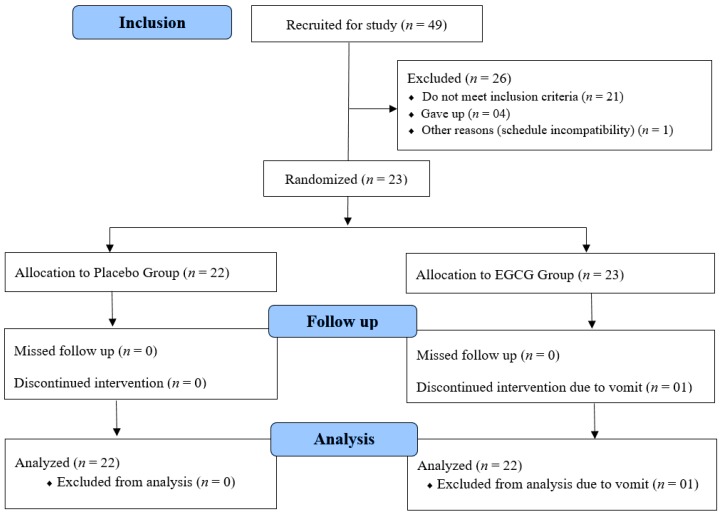
Study flowchart.

Twenty-three volunteers started the collection. One that was supplemented with EGCG showed vomiting and was excluded from the analysis, as presented in Figure 2. The 22 volunteers who completed the study were healthy women, aged 24.41 ± 0.56 years, BMI 21.11 ± 0.40 kg/m^2^ and fat percentage of 33.51 ± 1.38%. Sample characteristics are presented in Table 1.

The evaluation of baseline showed no difference between the EGCG group and the placebo group, as presented in Table 2. This shows that there was no residual effect carryover for any of the variables of study.

### 3.1. Gastric Emptying

**Figure 3:**
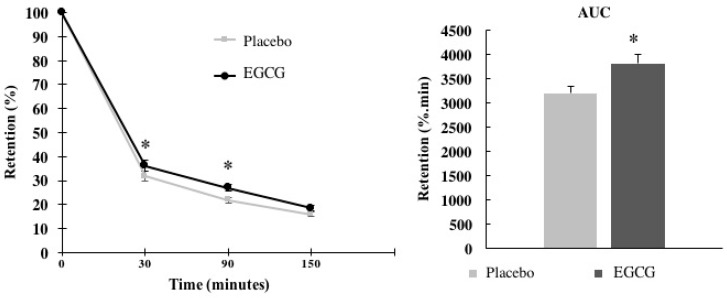
Relative gastric retention (%). Relative gastric retention variations and the area under the curve between treatments (EGCG × placebo) were assessed using the two-way ANOVA (adjusted to individual variation). Values are mean ± SEM. Differences found are highlighted with * (*p* < 0.05).

It was possible to verify the treatment effect on gastric retention (%) (*p* < 0.001). The interaction time × treatment differed at 30 min (T_30_ placebo: 31.34 ± 1.85%; EGCG: 36.2 ± 2.23%; *p* = 0.008) and 90 min (T_90_ placebo: 21.8 ± 1.06%; EGCG: 27.01 ± 1.56%; *p* = 0.005), both with large effect size (0.81 and 0.87, respectively). The AUC for relative gastric retention (%) also differed between interventions (*p* = 0.001), with a large effect size (1.12), as shown in Figure 3.

Regarding the emptying rate of calories per minute, there was a medium effect size for slower calorie flow in the EGCG group than the placebo group (placebo: 1.57 ± 0.02 kcal/min; EGCG: 1.52 ± 0.02 kcal/min; *p* = 0.053, effect size = 0.71).

### 3.2. Appetite Perceptions

#### 3.2.1. Hunger

**Figure 4:**
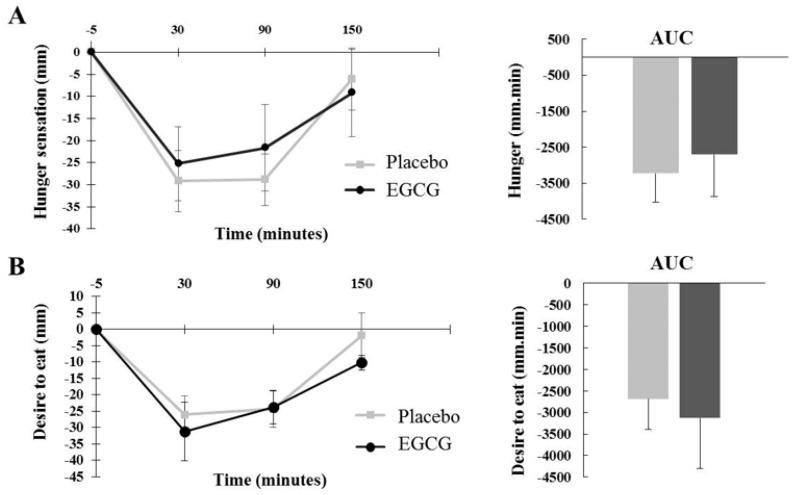

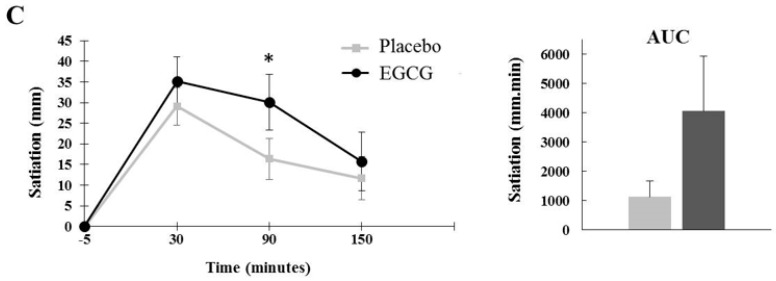
Variations in hunger sensation (**A**), desire to eat (**B**) and satiation (**C**)—Visual Analogue Scale (VAS). Perception of hunger, desire to eat and satiation variations assessed with VAS and the area under the curve (AUC) between treatments (placebo × EGCG) were evaluated using the two-way ANOVA (adjusted to individual variation). Values are mean ± SEM. Differences found are highlighted with * (*p* < 0.05).

Although there were differences regarding hunger sensation between the time periods assessed (*p* = 0.002), there was no difference between supplementation (*p* = 0.576) or the interaction time × treatment at the analyzed moments. Thus, there was no difference in the AUC (*p* = 0.646), as shown in Figure 4A.

#### 3.2.2. Desire to Eat

Regarding desire to eat, no difference was found between the supplementations (*p* = 0.345). The interaction time × treatment was not different between groups at any of the intervals (*p* > 0.05), as well as the AUC (*p* = 0.657), as shown in Figure 4B.

#### 3.2.3. Fullness (Satiation)

When we evaluated satiation (feeling of a full stomach), we observed significant findings in relation to time (*p* = 0.001) and treatment (*p* = 0.041). Although the interaction time × treatment at 30 min (*p* = 0.368) and 150 min (*p* = 0.539) was not different between groups, at 90 min, supplementation with EGCG was effective in increasing satiation compared to placebo with mean effect size (T_90_ placebo: 16.36 mm ± 5.04; EGCG: 30.09 ± 6.7 mm; *p* = 0.041, effect size = 0.62). However, there was no difference in the AUC (*p* = 0.115), as shown in Figure 4C.

### 3.3. Blood Glucose, Insulin, Leptin and Adiponectin Responses

#### 3.3.1. Glucose and Insulin

**Figure 5:**
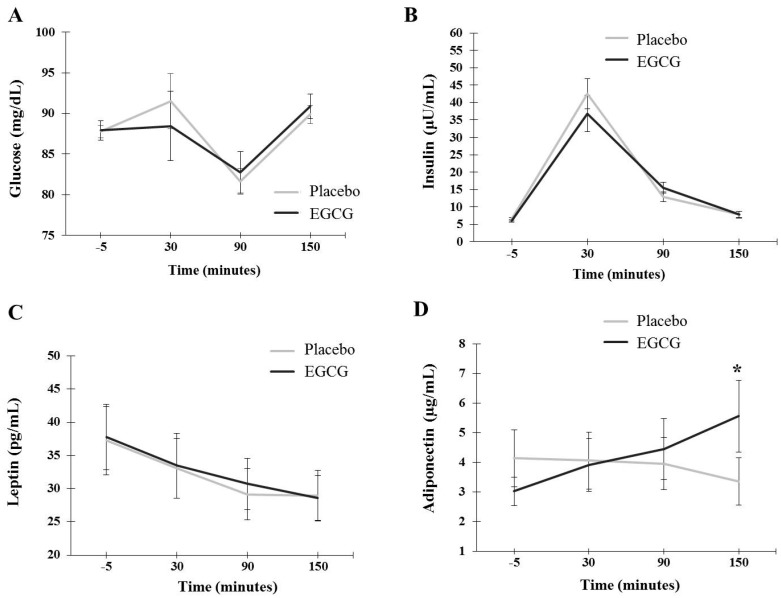
Plasma concentrations of glucose (**A**), insulin (**B**), leptin (**C**) and adiponectin (**D**). Plasma concentrations of glucose, insulin, leptin and adiponectin between treatments (placebo × EGCG) were assessed using the two-way ANOVA (adjusted to individual variation). Values are mean ± SEM. Differences found are highlighted with * (*p* < 0.05).

Blood glucose concentrations did not differ between the treatments (*p* = 0.802) and the interaction time × treatment at any time evaluated, as presented in Figure 5A. Regarding insulin concentrations, there was no difference between the treatments (*p* = 0.586), with medium effect size at T_30_ effect size = 0.53 (Figure 5B). 

#### 3.3.2. Leptin and Adiponectin.

Regarding leptin, there was no difference between the treatments (*p* = 0.674) and the interaction time × treatment for any time evaluated (*p* > 0.05), as presented in Figure 5C. The plasma concentrations of adiponectin showed no difference in the interaction time × treatment between the EGCG and placebo groups at 30 min (*p* = 0.811) and 90 min (*p* = 0.414). At 150 min, the volunteers supplemented with EGCG had higher production when compared to placebo, with large effect size (T_150_ placebo: 3.36 ± 0.8 µg/mL; EGCG: 5.56 ± 1.21 µg/mL; *p* = 0.004, effect size = 1.14), as presented in Figure 5D.

## 4. Discussion

In this study, we evaluated the acute action of EGCG (752 mg) on appetite response in three ways: gastric emptying through ultrasound (a noninvasive and reproducible technique); perceptions of hunger, desire to eat and fullness through use of VAS and blood hormones related to appetite control (insulin, leptin and adiponectin); and glucose concentrations. Our main findings were that acute EGCG supplementation induced higher gastric relative volume in 30 min, which resulted in a higher AUC gastric retention. Although there was no difference in hunger or desire to eat, satiation was higher at 90 min and secretion of adiponectin at 150 min after EGCG intake. Regarding gastric emptying, EGCG was able to retain more fluid in the stomach after 30 min and 90 min of the supplementation. Furthermore, when assessing the whole intervention period (150 min), the AUC revealed higher gastric retention in the group supplemented with EGCG.

Gastric emptying is one of the main factors responsible for the increase in blood glucose concentrations in the first 30 min postprandial, and individual glycemic response after oral ingestion is one of the mechanisms to control the release of gastric contents [15]. Therefore, there is an interdependent relation between the two. The faster the deflation, the higher the increase of initial post-meal blood glucose [15,16]. This study showed that the glycemic response did not differ between groups. However, there was a medium effect size on insulin release after 30 min of mixed-nutrient drink intake only when placebo was offered, which could mean that EGCG group’s glycemic control was induced by lower insulin production, leading to a physiological response of slowing gastric emptying.

The rates of carbohydrate, protein and lipid gastric emptying vary individually with approximately 2 mL/min or 1 to 4 kcal/min in healthy individuals [9]. The standard liquid meal offered in this study consisted of a mixture of macronutrients. We observed that when supplemented with EGCG, the output of calories presented a mean of 1.52 kcal/min and when given the placebo, it was 1.57 kcal/min, both of which were within the normal range for healthy individuals [9]. However, the intervention with EGCG showed a medium effect size for emptying calories more slowly.

The distension of the proximal portion of the stomach generates short-term information to the central nervous system (CNS) about the amount of ingested nutrients and transmits satiation perceptions during the meal (fullness) [17]. The volunteers ate the same volume and nutrients in both interventions and, despite the increased gastric retention with EGCG after 30 min and 90 min, there was an increased feeling of a full stomach (satiation) only at 90 min. The analysis of the AUC of satiation throughout the study (150 min) showed no difference between the interventions. Thus, unlike previous studies [9,17,18], the longest distension time of the stomach wall and the slower emptying of the bolus into the intestine did not favor the feeling of fullness or satiation in the group that received EGCG. There was also no change regarding hunger or desire to eat in any of the evaluated times.

Studies have shown that the way the meal is offered may interfere with appetite [19,20,21]. Foods consumed with the same number of calories, but in different forms (liquid or solid), affect satiety differently. Liquids tend to produce less satiety compared to solid foods [22,23,24]. Although we offered the amount of nutrients and calories often consumed by the participants, most women had the habit of eating salty and solid preparations for breakfast, and the offer of a sweet liquid mixed-nutrient drink may have affected hunger and desire to eat. Thus, the VAS may reflect individual preferences and cause inconsistencies in the subjective evaluation, since previous experience and the food environment generate distinct individual habits and preferences for salty or sweet flavors [25]. Additionally, the fact that women demonstrated a high amount of body fat, especially abdominal, may have affected the taste and smell perception of the test meal, as in an earlier study that found negative association between gustatory and olfactory function with visceral fat quantity [26].

Regarding the analysis of adipose tissue hormones that control appetite, there was no difference in the production of leptin between the groups at any time. A previous study with green tea catechin supplementation, including 843 mg of EGCG, in postmenopausal overweight women also found no change in leptin, ghrelin and adiponectin concentrations after supplementation with catechins [27]. However, in our study, there was a gradual increase in the release of adiponectin in the group that received EGCG.

Adiponectin acts by improving insulin sensitivity through muscle glucose uptake stimulation and hepatic glucose production inhibition [28]. However, adiponectin released 30 min after supplementation showed no difference between the groups and may not have been responsible for better insulin sensitivity in the EGCG group at that evaluated time. Studies using the green tea extract including EGCG in culture cells [29,30], animals [31] and humans [32] show improved insulin sensitivity by stimulating the glucose uptake pathway, insulin signaling, inhibition of hepatic gluconeogenesis and stimulation of GLP-1. However, the literature is controversial and some studies, including a meta-analysis, found no difference in the improvement of glucose metabolism [33,34].

In addition to the peripheral action, adiponectin has receptors in the hypothalamus and acts by controlling food intake. Adiponectin with low and medium molecular weight (trimers and hexamers) act by increasing appetite and stimulating food intake acutely in the fasting period by stimulating protein kinase activated by AMP (AMPK) via AdipoR1receptor [35]. Thus, the highest concentration of adiponectin in the EGCG group at the end of the study (150 min) may have influenced the appetite, increasing hunger and desire to eat and reducing the satiation of the volunteers. However, in our study, we were not able to evaluate the fractions with different molecular weights of adiponectin and, thus, we cannot say with certainty that the increase in plasma adiponectin concentration was able to overcome the barrier and enter the cerebrospinal fluid to take action in CNS and modify appetite. Furthermore, the design of this study cannot explain the reason why adiponectin increases at 150min after EGCG supplementation.

In general, the EGCG dose tested (752 mg) was well tolerated. Two volunteers reported mild nausea after 90 min of capsules ingestion. Only one participant who received capsules with EGCG presented vomiting after 90 min of ingestion and had to be excluded from the study. The dose of combined green tea catechins used in the studies ranged from 141 mg/day to 1207 mg/day [7]. A clinical study conducted with healthy subjects [36] tested the toxicity of 800 mg/day of isolated EGCG for 4 weeks and observed that there were no serious symptoms or adverse effects, rendering it a safe dose for ingestion. The EGCG dose tested in this study (752 mg) is equivalent to approximately 9 cups of green tea. Thus, this amount would be obtained by supplementation.

As positive aspects, we can point out that this study suggests new evidence of EGCG action and was the first to show that EGCG acts in the slowing of gastric emptying. In addition, the crossover type of design enabled the exclusion of the individual physiological response variation. In addition to that, standardization of the meal prior to the experiment (dinner) may have reduced the food bias and ensured that the alterations were due to the action of EGCG.

The limitation of this study was the need to have offered a preparation of liquid consistency, which differs from the usual eating habits of the women studied. This fact may have influenced the appetite perceptions of VAS, especially hunger and desire to eat. Also, we did not evaluate gastrointestinal hormones related to appetite, such as ghrelin and GLP-1, which could affect the findings of our study. However, further studies are encouraged to investigate these hormonal responses.

## 5. Conclusions

Acute EGCG supplementation is able to delay gastric emptying in healthy women to a small but statistically significant extent. Further studies with larger intervention periods and in overweight individuals may help to elucidate the influence of gastric slowing on appetite regulation and long-term food intake.

## Figures and Tables

**Table 1:** Anthropometric, body composition and habitual food intake characteristics.

Parameters	Mean	SEM
Anthropometry		
Body weight (kg)	56.63	±1.46
Height (m)	1.64	±0.01
Body mass index (kg/m)	21.11	±0.40
Waist circumference (cm)	68.81	±1.09
Hip circumference (cm)	93.05	±1.88
Sagittal abdominal diameter (cm)	16.69	±0.50
Body composition		
Total fat (%)	33.51	±1.38
Torso fat (%)	29.55	±1.63
Android fat (%)	34.9	±2.04
Gynoid fat (%)	47.62	±1.35
Habitual food intake		
Calories (kcal/day)	1876.59	±117.11
Calories (kcal/kg/day)	33.71	±2.41
Total proteins (g/day)	82.6	±5.84
Total proteins (g/kg/day)	1.47	±0.11
Total proteins (%)	18.14	±1.26
Total carbohydrates (g/day)	201.82	±15.61
Total carbohydrates (%)	43.07	±2.09
Total lipids (g/day)	82.02	±6.83
Total lipids (%)	38.75	±1.82
Dietary fiber (g/day)	19.1	±2.22
Water ingestion—pure water (L/day)	1.84	±0.15

The data represented mean ± SEM.

**Table 2:** Stomach ultrasound parameters, Visual Analogue Scale and biochemical analysis at baseline (- 5 min).

Parameters	Placebo Group	EGCG Group	*p* Value
	Mean ± SEM	Mean ± SEM	
Absolute gastric value (mL)	349.58 ± 3.53	348.51 ± 3.46	0.859
Hunger sensation (mm)	64.05 ± 6.61	61.14 ± 5.33	0.628
Desire to eat (mm)	70.32 ± 6.04	68.86 ± 6.50	0.817
Fullness-satiation (mm)	21.23 ± 4.75	15.14 ± 3.33	0.178
Glycemia (mg/dL)	87.77 ± 0.79	87.91 ± 1.2	0.965
Insulin (μU/mL)	6.49 ± 0.46	6.03 ± 0.44	0.887
Adiponectin (µg/mL)	4.14 ± 0.96	3.02 ± 0.48	0.067
Leptin (pg/mL)	37.24 ± 5.14	37.76 ± 4.95	0.840

Measured values at starting moment (- 5 min) between treatments (placebo × EGCG) were compared using the two-way ANOVA (adjusted to individual variation). The data represented mean ± SEM.

## Supplementary Materials

The following are available online at http://www.mdpi.com/2072-6643/10/8/1122/s1, Table S1: Liquid test meal nutritional characteristics (mixed-nutrient drink), Table S2: Standard solid meal nutritional characteristics (dinner).
