# Supplementary material for: Acute Epigallocatechin 3 Gallate (EGCG) Supplementation Delays Gastric Emptying in Healthy Women: A Randomized, Double-Blind, Placebo-Controlled Crossover Study
Source: Nutrients. 2018 Aug 20;10(8):1122. doi: 10.3390/nu10081122 (PMC6115961; doi:10.3390/nu10081122)
Supplement: Supplementary file 1 [file nutrients-10-01122-s001.pdf]

nutrients

SUPPLEMENTARY MATERIAL

Table S1. Liquid test meal nutritional characteristics (mixed-nutrient drink).

Variables		Quantidade
Foods		
	Whole milk (mL)	180
	Papaya (g)	30
	Banana (g)	50
	Whole milk powder (g)	20
	Refined sugar (g)	05
Nutritional calculation		
	Calories (kcal)	279.44
	Caloric density (kcal/mL)	0.95
	Total proteins (%)	19.65
	Total carbohydrates (%)	60.64
	Total lipids (%)	19.71

The data represented mean ± SEM.

Table S2. Standard solid meal nutritional characteristics (dinner).

Variables		Quantity
Foods		
	Cooked sweet potato (g)	220
	Cooked chicken breast shredded (g)	120
	Cooked carrot (g)	20
	Tomato (g)	40
	Extra virgin olive oil (mL)	08
	Parmesn cheese (g)	10
	Raw onion (g)	05
	Garlic (g)	01
Nutritional calculation		
	Calories (kcal)	484.33
	Total proteins (g)	43.36
	Total proteins (%)	35.81
	Total carbohydrates (g)	44.49
	Total carbohydrates (%)	36.74
	Total lipids (g)	14.77
	Total lipids (%)	27.45

The data represented mean ± SEM.
